# Nucleotide excision repair deficiency in melanoma in response to UVA

**DOI:** 10.1186/s40164-016-0035-4

**Published:** 2016-02-24

**Authors:** Heather C. Murray, Vicki E. Maltby, Doug W. Smith, Nikola A. Bowden

**Affiliations:** School of Biomedical Sciences and Pharmacy, University of Newcastle, Hunter Medical Research Institute, University Dr, Callaghan, NSW 2308 Australia

**Keywords:** Melanoma, UVA, DNA repair, CPDs, Global genome repair, Nucleotide excision repair, XPC, DDB1, DDB2, p53

## Abstract

**Background:**

The causative link between UV exposure and melanoma development is well known, however the mechanistic relationship remains incompletely characterised. UVA and UVB components of sunlight are implicated in melanomagenesis; however the majority of studies have focused on the effects of UVB and UVC light. Interestingly, melanoma tumour sequencing has revealed an overrepresentation of mutations signature of unrepaired UV-induced DNA damage. Repair of UVA-induced DNA damage is thought to occur primarily through the Nucleotide Excision Repair (NER) pathway, which recognises and repairs damage either coupled to transcription (Transcription Coupled Repair; TCR), or through global genome scanning (Global Genome Repair; GGR). Current literature suggests NER is deficient in melanoma, however the cause of this remains unknown; and whether reduced NER activity in response to UVA may be involved in melanoma development remains uncharacterised. In this study we aimed to determine if melanoma cells exhibit reduced levels of NER activity in response to UVA.

**Methods:**

Melanocyte and melanoma cell lines were UVA-irradiated, and DNA damage levels assessed by immunodetection of Cyclobutane Pyrimidine Dimer (CPD) and (6-4) Photoproduct [(6-4)PP] lesions. Expression of NER pathway components and p53 following UVA treatment was quantified by qPCR and western blot.

**Results:**

UVA did not induce detectable induction of (6-4)PP lesions, consistent with previous studies. Repair of CPDs induced by UVA was initiated at 4 h and complete within 48 h in normal melanocytes, whereas repair initiation was delayed to 24 h and >40 % of lesions remained in melanoma cell lines at 48 h. This was coupled with a delayed and reduced induction of GGR component XPC in melanoma cells, independent of p53.

**Conclusion:**

These findings support that NER activity is reduced in melanoma cells due to deficient GGR. Further investigation into the role of NER in UVA-induced melanomagenesis is warranted and may have implications for melanoma treatment.

## Background

Melanoma derives from the malignant transformation of melanocytes [1, 2]. Cutaneous melanoma, or melanoma of the skin, represents the most common form of the disease; accounting for more than 90 % of cases [3]. Cutaneous melanoma tumours are commonly non-responsive to standard DNA-damaging chemotherapeutic agents. Targeted therapies to the BRAF V600E mutation present in 30–60 % of metastatic melanomas [4, 5] and immune-therapy based approaches display the highest treatment response rates [6, 7].

UV light exposure is estimated to be causative of more than 90 % of cutaneous melanoma cases [8]. Despite this, the mechanism by which UV light leads to melanoma development remains incompletely characterised. The UV spectrum is subdivided into UVC (100–280 nm), UVB (280–315 nm), and UVA (315–400 nm) light but UVA constitutes approximately 95 % of environmental UV exposure with UVB making up the remaining 5 % [9, 10]. The relatively lower energy UVA rays penetrate deepest into the skin; therefore, UVA is estimated to account for 99 % of UV that melanocyte skin cells are naturally exposed to [11, 12].

Despite its environmental abundance, the UVA component of the UV spectrum is understudied. Until recent years UVA exposure was considered non-harmful, with UVA doses in the range of 100,000 J/m^2^ required to produce comparable cellular effects as UVB doses approximately 100 fold lower, and UVC doses 10,000 fold lower [13–15]. Epidemiological assessments of human melanoma incidence as a function of environmental UV exposure have reported that UVA exposure is an independent risk factor for melanoma development [16–18]. Consistent with this, UVA tanning bed use has been associated with an increased risk of developing melanoma [19–22]. The International Agency for Research in Cancer (IARC) now recognises UVA as a class I carcinogen [23].

UV radiation induces two main forms of DNA damage: pyrimidine dimers and oxidative base damage. Pyrimidine dimers are formed by direct absorption of UV energy by DNA, leading to the formation of covalent bonding between contiguous pyrimidine bases [24, 25]. This results in either a Cyclobutane Pyrimidine Dimer (CPD) or (6-4) Pyrimidine Pyrimidone [(6-4)PP] structure [26]. CPD and (6-4)PP lesions both distort the helical structure of DNA, forming substrates for the DNA repair process, known as nucleotide excision repair (NER) [27]. Left unrepaired, C > T transition mutations often result due to the misincorporation of Adenine opposite the lesion. As such, C > T transitions are considered UV ‘signature’ mutations [28].

NER is a vital DNA repair process that detects and repairs lesions that cause both chemical alteration and structural distortion of the DNA helix [27] such as CPDs, 6-4PPs and platinum-based chemotherapy induced DNA crosslinks. The NER pathway possesses two different mechanisms for detection of DNA damage; Transcription Coupled Repair (TCR) and Global Genome Repair (GGR; for extensive review, see [29–32]). TCR detects lesions in transcribed sections of the genome; triggered when a lesion inhibits transcription elongation by RNA polymerase II. GGR detects lesions across the whole genome, including non-transcribed regions. Upon lesion detection by either the TCR or GGR arm, repair proceeds via a final common pathway [33]. Pathogenic mutations in the GGR components XPC and DDB2 (XPE) result in xeroderma pigmentosum (XP) a disease characterised by increased UV-sensitivity and skin cancer incidence. Conversely, mutation in TCR genes ERCC8 (CSA) and ERCC6 (CSB) result in Cockayne’s syndrome that is characterised by neurological abnormalities but no increase in skin cancer incidence. Some NER proteins, particularly the GGR damage recognition proteins, can decide a cell’s fate by triggering the initiation of the repair pathway or by signalling apoptosis [34]. Therefore, if the GGR pathway is defective, neither DNA repair nor apoptosis occurs, resulting in a cancer cell containing high levels of UV-induced mutations that does not undergo apoptosis, both of which are features of melanoma [35].

The majority of research characterising the NER response to UV has been performed using UVB and UVC. CPD lesion removal following UVA has been assessed in keratinocytes [36] and human skin [37], however the NER response to UVA has not been characterised at the transcript or protein level, for any cell type. The current literature suggests that NER, and in particular the global genome repair (GGR) damage recognition sub-group [38, 39], may be deficient in melanoma. The effect this has on repair of UVA-induced DNA damage in melanoma is unknown. In this study we aimed to determine if melanoma cells exhibit reduced levels of NER activity in response to UVA. We quantified expression of the entire NER pathway, and confirmed altered activity of the GGR components XPC, DDB1, DDB2, and p53 following UVA treatment; and CPD and (6-4)PP lesion repair following UVA irradiation in melanoma compared to normal melanocytes.

## Methods

### Cell culture

The non-transformed Human Epidermal Melanocyte adult Lightly Pigmented cell strain HEMaLP was purchased from Life Technologies. Metastatic melanoma cell line Mel-RM and primary melanoma cell lines Sk-Mel-28, MM200, and Me4405 were derived as described previously [40, 41]. The mutation status of these cell lines for p53 are: Mel-RM wild-type, Sk-mel-28 p53 R273H, MM200 p53 wild-type and Me4405 p53 null.

Cells were maintained in standard culture conditions at 5 % CO_2_, 37 °C. Melanocytes were cultured in Medium 254 containing human melanocyte growth supplement (HMGS; Life Technologies). Melanoma cells were cultured in 1× complete DMEM (Gibco, Life Technologies;) supplemented with 10 % FBS (SAFC Biosciences, Sigma-Aldrich), All cell lines were routinely confirmed to be free of mycoplasma contamination. Authentication of cell lines was performed by the Australian Genome Research Facility using the GenePrint 10 system.

### UVA treatment

Cells at confluence were UVA-irradiated using a Grobel Irradiation Chamber (Dr. Gröbel UV-Elektronik GmbH, Germany) at 100 kJ/m^2^. This is a physiologically relevant dose of UVA; attainable in approximately an hour of summer sun exposure [10]. 100 kJ/m^2^ UVA was also sufficient to induce a low level of cell death in the majority of cell lines across 48 h following irradiation (data not shown).

### DNA damage detection

CPD and (6-4)PP lesions were quantified using an infrared immunodetection microplate assay, as previously described [42] with some modifications. Cells were grown to confluence in 96 well plates, irradiated with UVA and incubated for the required time period (0, 1, 4, 24, or 48 h). Cells were then fixed and permeabilised by incubation in prechilled 1:1 methanol/acetone, for 10 min at −20 °C. Plates were washed twice in PBS, followed by denaturation of cellular DNA by incubation in hydrochloric acid (HCl) for 30 min at room temperature. DNA denaturation with 2 M HCl was determined to be optimal for CPD detection, and 0.5 M HCl for (6-4)PP detection. Following two washes in PBS-TB (0.1 % Tween 20, 1 % BSA), blocking PBS-TB for 90 min at room temperature, cells were probed for DNA damage levels by overnight incubation at 4 °C with anti-(6-4)PP antibody (1:1000; clone KTM50, Kamiya Biomedical Company) or anti-CPD antibody (1:1000; clone KTM53) diluted in PBS-TB.

Plates were then washed five times in PBS-TB and incubated with secondary antibody (1:800 IRDye goat-anti-mouse 800CW; LI-COR) for 1 h at room temperature. Cells were concurrently stained with the infrared non-specific cellular stain CellTag700 (1:500; LI-COR), to enable normalisation of the damage signal to cell number. Following five washes in PBS-T, plates were scanned on a LI-COR Odyssey CLx infrared imager. Quantification was performed using Image Studio (LI-COR). Standard curves were run to ensure CPD, (6-4)PP and CellTag700 signal was measured in the linear range (data not shown).

### NER transcript expression

RNA was extracted using TRIzol reagent (Invitrogen) as per manufacturer’s instructions, or in combination with an RNeasy kit (Qiagen). Melanin contaminated RNA samples were purified using OneStep PCR inhibitor removal kits (Zymo Research). RNA was reverse transcribed using High Capacity cDNA Reverse Transcription Kits (Applied Biosystems). A standardised amount of RNA (2ug) was reverse transcribed for all samples. Resultant cDNA was diluted to a 1:20 working concentration.

The expression of 13 NER transcripts, p53 (Hs00153340_m1) and two housekeeping (endogenous control) transcripts GAPDH (4326317E) and β-actin (4326215E) were quantified using Taqman Gene Expression Assays (Applied Biosystems) and a ViiA7 (Applied Biosystems). NER transcripts assays: XPC (Hs01104206_m1), DDB1 (Hs00172410_m1), DDB2 (Hs00172068_m1), CSA/ERCC8 (Hs01122123_m1), CSB/ERCC6 (Hs00972920_m1), XPD/ERCC2 (Hs00361161_m1), XPB/ERCC3 (Hs01554450_m1), XPG/ERCC5 (Hs00164482_m1), XPF/ERCC4 (Hs00193342_m1), ERCC1 (Hs01012158_m1), XPA (Hs00166045_m1), RPA1 (Hs00161419_m1), RPA2 (Hs00358315_m1).

NER transcript expression was normalised relative to the endogenous control *GAPDH*. Of the housekeeping genes quantified, *GAPDH* demonstrated the lowest variability in expression. *GAPDH* expression was not modulated by treatment; this was verified by determining the ratio of expression compared to that of *β*-*actin*. Average *GAPDH/β*-*actin* ratio across all samples was 0.90 ± 0.002 (average ± SEM).

Relative expression (RE) was calculated by 2^−∆Ct^ [43]. Induction (fold change) of expression from baseline was calculated by dividing by the baseline RE value; induction at baseline was by definition set at 1. Induction values less than 1 were converted to negative values. RE and induction results represent the mean of triplicate determinations of three independent experiments, ±SEM. For further analysis melanoma cell lines were grouped by p53 status, and averaged results for p53 wildtype (p53+; MM200 and Mel-RM) and p53 mutant/null (p53−; SK-Mel-28 and Me4405 respectively).

### NER protein expression

Whole cell extracts were prepared in, RIPA buffer (50 mM Tris–HCl pH 7.5, 150 mM NaCl, 1 % NP-40, 0.5 % sodium deoxycholate, 0.1 % SDS) containing complete mini protease inhibitors (Roche). Cells were lysed, insoluble material was pelleted by centrifugation (5 min, 12,000 rpm) and the supernatant collected. Protein quantification was performed by bicinchoninic acid (BCA) assay, as per manufacturer’s instructions (Thermo-Scientific).

XPC (Santa Cruz A-5, 1:250), DDB1 (Invitrogen ZD001, 1:1000), DDB2 (Abcam 2246C4, 1:50), and p53 (Abcam DO-1, 1:500) were assessed by SDS-PAGE followed by western blot using GAPDH (Abcam EPR6256, 1:1000) as a loading control. p53 and GAPDH primary antibodies were detected using IRDye labelled goat-anti-mouse (LI-COR) and goat-anti-rabbit (LI-COR) respectively. Blots were then visualised on a LI-COR Odyssey CLx infrared imager. DDB1, DDB2, and XPC were detected using goat-anti-mouse HRP-conjugated secondary antibody (1:15,000; Biorad) and developed by incubation in SuperSignal West Femto Chemiluminescent substrate (ThermoFisher) and developed on a FujiFilm LAS3000 imager (FujiFilm Medical Systems). All blot images underwent densitometry analysis using Image Studio v3.1 (LI-COR). Quantification was performed by normalisation to GAPDH and expressed as fold induction from baseline. Standard curves were run for all proteins of interest, to ensure detection was in the linear range (data not shown).

### Statistical analysis

Non-parametric Mann–Whitney U tests were performed with SPSS software (IBM corporation), and obtained p values were corrected for multiple comparisons using the Holm-Sidak approach. p < 0.05 was considered significant.

## Results

### Melanoma cells are slower to repair CPD lesions caused by UVA damage

**Fig. 1:**
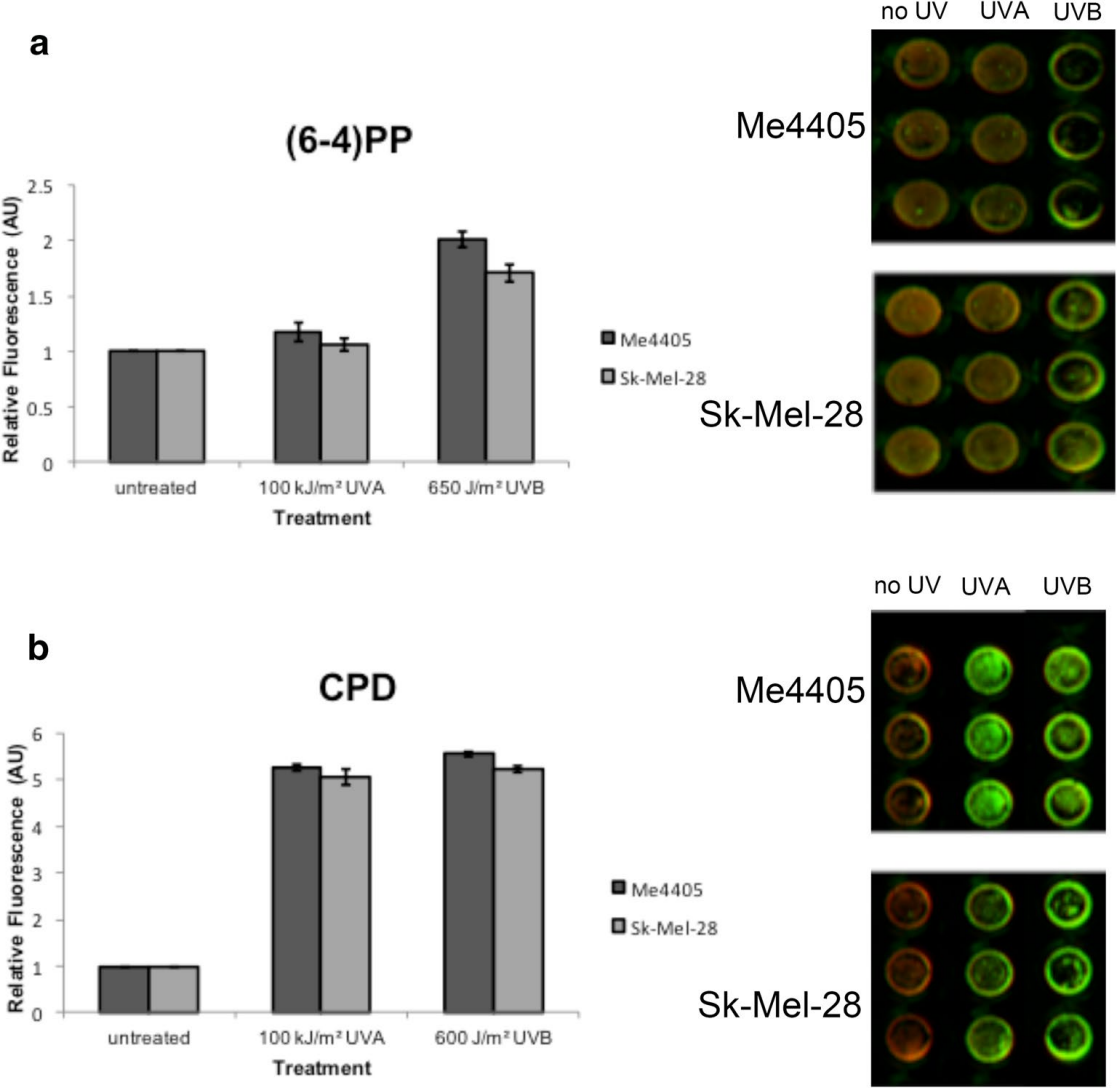
Immunodetection of (6-4)PP and CPD induction in response to UVA and UVB. Melanoma (Me4405, Sk-Mel-28) cell lines were irradiated with UVA or UVB, and DNA damage lesions were quantified at 0 h. **a** (6-4)PP and **b** CPD quantitation and corresponding raw images. Signal was normalised to cell number; *points* mean of three independent experiments, *bars* SEM. Images depict cell stain signal (*red*, 700 nm channel) overlaid with the DNA damage signal (*green*, 800 nm channel)

To quantify DNA damage induced by UVA cell lines were irradiated with a solar available dose of UVA (100 kJ/m^2^) and antibody-mediated detection of CPD and (6-4)PP lesions was used for quantification. Consistent with previous reports, 100 kJ/m^2^ UVA did not induce detectable (6-4)PP (Fig. 1a) but did induce CPD lesions, at a similar level to 600 J/m^2^ UVB (Fig. 1b).

**Fig. 2:**
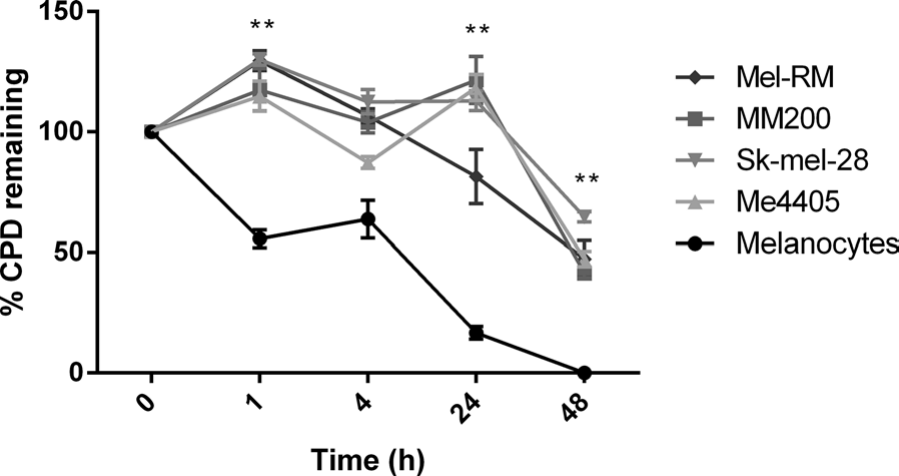
CPD repair after UVA in melanocytes and melanoma. Melanocyte (HEMaLP) and melanoma cell lines (MM200, Sk-Mel-28, Me4405, Mel-RM) were irradiated with 100 kJ/m^2^ UVA, and CPD lesions quantified following irradiation. Results are shown as a percentage of 0 h. *Bars* mean of four independent experiments ± SEM. Significance determined by Mann–Whitney U test for all melanoma cell lines compared to melanocytes (HEMaLP) *p < 0.01

Melanocytes completed repair of CPD lesions within 48 h following exposure to 100 kJ/m^2^ UVA, with almost 50 % of repair occurring by 1 h, followed by the remainder of repair occurring after 4 h (Fig. 2). All the melanoma cell lines displayed a higher level of CPD lesions at all time points following UVA exposure when compared to melanocytes, reaching significant difference at 1 (p = 0.02), 24 (p = 0.02) and 48 h (p = 0.008). In all of the melanoma cell lines the majority of repair occurred after the 24 h time point, with more than 40 % of CPD lesions remaining at 48 h in all melanoma cell lines (Fig. 2). Between melanoma cell lines, no significant differences in repair rate were observed.

### Melanoma cells have delayed expression of GGR in response to UVA damage

To determine if NER deficiency was responsible for the lack of CPD repair in melanoma the relative expression of 13 NER pathway mRNA transcripts, and the NER regulator *p53*, were subsequently quantified. Of these, a marked difference between melanocyte and melanoma cell lines was identified for *p53* and the three GGR transcripts *XPC*, *DDB1*, and *DDB2.* The remainder of the NER pathway transcripts showed no consistent changes in expression between melanocytes and melanoma (Table 1).

**Table 1:** Relative expression of Transcription coupled repair (TCR) and nucleotide excision repair (NER) transcripts in melanocytes and melanoma cell lines following UVA

Gene	Cell Type	Time (h)									
		0		1		4		24		48	
		RE	P value	RE	P value	RE	P value	RE	P value	RE	P value
Transcription coupled repair (TCR)											
*ERCC6*	Melanocytes	0.0883	NS	0.0000	<0.001	0.1079	NS	0.0236	<0.001	0.3084	NS
	Melanoma	0.1345		0.1003		0.1534		0.1715		0.2396	
*ERCC8*	Melanocytes	0.0893	NS	0.0000	<0.001	0.1026	NS	0.0251	<0.001	0.1102	NS
	Melanoma	0.1707		0.0586		0.0780		0.0482		0.0980	
Nucleotide excision repair (NER)											
*XPA*	Melanocytes	0.1288	0.03	0.2592	<0.001	0.5513	<0.001	0.1530	NS	0.5681	NS
	Melanoma	0.0924		0.1161		0.1447		0.2406		0.3585	
*RPA1*	Melanocytes	0.4713	0.02	0.3281	<0.001	1.1675	NS	0.9926	0.038	4.1019	NS
	Melanoma	2.0848		1.3489		0.9849		2.4304		2.1699	
*RPA2*	Melanocytes	0.2883	0.002	0.4576	0.002	1.3346	NS	0.4085	<0.001	1.5100	NS
	Melanoma	1.7746		1.2359		1.2400		1.9103		1.6426	
*ERCC1*	Melanocytes	1.1466	NS	1.2102	NS	4.1937	<0.001	6.4460	<0.001	3.0374	0.04
	Melanoma	1.1916		1.4276		1.5437		1.5859		2.2263	
*ERCC2*	Melanocytes	0.6308	NS	0.9042	0.007	1.7292	<0.001	0.8925	<0.001	1.1017	0.03
	Melanoma	0.5406		0.4764		0.3694		0.4094		0.5468	
*ERCC3*	Melanocytes	0.1390	<0.001	0.7994	NS	0.8556	<0.001	0.2262	<0.001	1.0353	0.05
	Melanoma	0.4889		0.4753		0.3632		0.6632		0.6733	
*ERCC4*	Melanocytes	0.1018	NS	0.0000	<0.001	0.1832	0.009	0.0000	<0.001	0.4600	NS
	Melanoma	0.1977		0.1656		0.1709		0.2446		0.3675	
*ERCC5*	Melanocytes	0.0537	0.001	0.0000	<0.001	0.1138	NS	0.0000	<0.001	0.4116	NS
	Melanoma	0.2245		0.2294		0.1160		0.2777		0.3699	

**Fig. 3:**
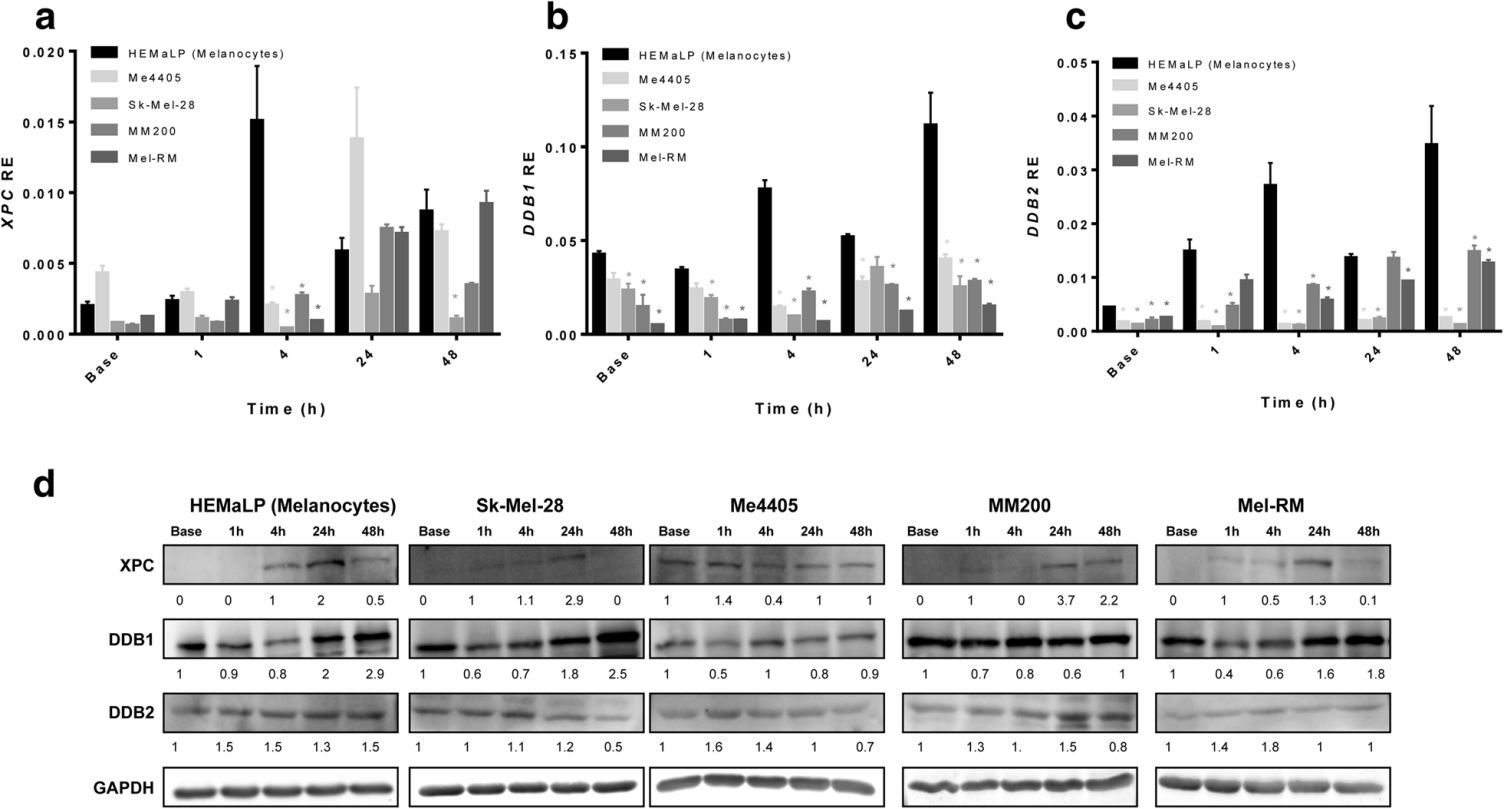
Expression of global genome repair XPC, DDB1, and DDB2 following UVA irradiation in melanocytes and melanoma. Cell lines were irradiated with 100 kJ/m^2^ UVA, then mRNA transcript relative expression (RE) at baseline and 1, 4, 24, and 48 h post-irradiation was determined. **a** RE of *XPC* mRNA transcript **b** *DDB1* mRNA transcript, **c** *DDB2* mRNA transcript. Significance compared to melanocytes (HEMaLP) determined by Mann–Whitney U test. *p < 0.05. *Points* mean of triplicate determinations of three independent experiments; *bars* ± SEM. **d** Western blot of XPC, DDB1 and DDB2 for all cell lines before and after UVA. *Blots* are representative of duplicate blots run for all proteins and cell lines

*XPC* transcript displayed a bimodal induction pattern, with expression peaking at 4 and 48 h (Fig. 3a) and was significantly higher in melanocytes compared to all melanoma cell lines, at 4 h following UVA exposure (Fig. 3a). Similarly, XPC protein peaked at 4 and 24 h (Fig. 3d). The XPC expression directly aligned with the majority of CPD repair occurring after 4 h in melanocytes (Fig. 2). In comparison, melanoma cells displayed a delayed response, with transcript expression peaking at 24 or 48 h (Fig. 3a) and XPC protein peaking at 24 h in all cell lines except Me4405 (Fig. 3d). The XPC expression in melanoma also reflected the CPD repair occurring predominantly after 24 h in all melanoma cell lines (Fig. 2).

*DDB1* transcript expression was significantly higher in melanocytes at all time points (Fig. 3b), particularly 4 and 48 h post-UVA. Following a slight decline 1 h post-UVA, DDB1 protein expression was increased in melanocytes in response to UVA, with maximal induction at 48 h. Two (Sk-Mel-28, Mel-RM) of four melanoma cell lines displayed a DDB1 protein induction profile comparable to this (Fig. 3d). Similarly, DDB2 transcript levels were significantly higher in melanocytes at almost all time points, in particular at 4 and 48 h (Fig. 3c). The same trend occurred at 24 h, but did not reach significance. DDB2 protein abundance was low in all cell lines, only slight increase were detected in each of the cell lines at various timepoints (Fig. 3d). In summary, XPC expression most accurately reflected the delay in CPD repair in melanoma.

### GGR deficiency in melanoma is independent of p53

**Fig. 4:**
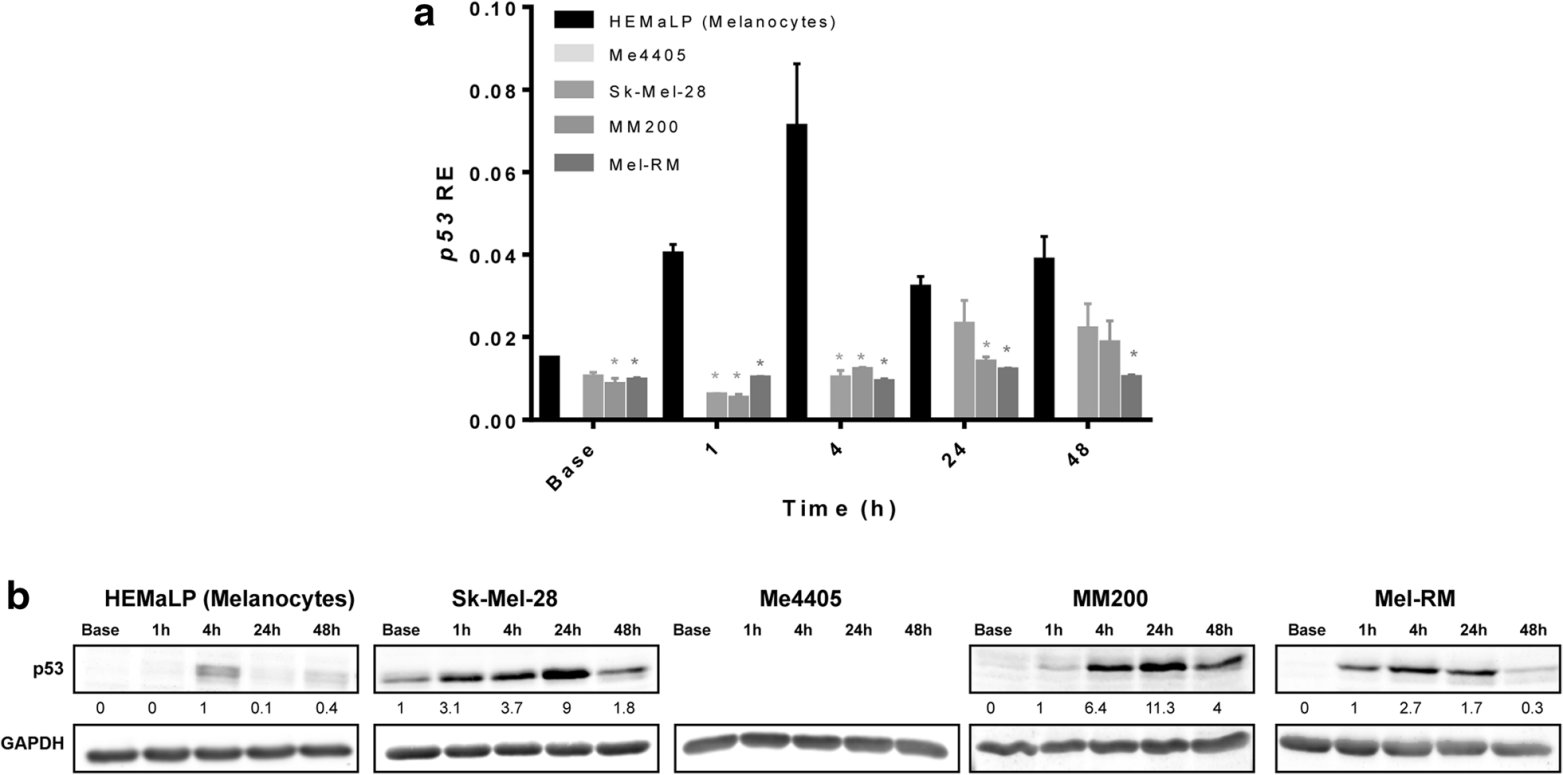
Expression of p53 following UVA irradiation in melanocytes and melanoma. Cell lines were irradiated with 100 kJ/m^2^ UVA, then mRNA transcript relative expression (RE) at baseline and 1, 4, 24, and 48 h post-irradiation was determined. **a** RE of *p53* mRNA transcript. Significance compared to melanocytes (HEMaLP) determined by Mann–Whitney U test. *p < 0.05. *Points* mean of triplicate determinations of three independent experiments; *bars* ± SEM. **b** Western blot of p53 for all cell lines before and after UVA. *Blots* are representative of duplicate blots run for all proteins and cell lines

p53 is known to directly regulate NER function, [44, 45]. However, XPC [46] and DDB2 [47] may also affect expression of p53, suggesting the presence of a feedback loop. In addition, XPC and DDB2 also function independently of p53 to induce apoptosis [48, 49]. To investigate the possible regulatory relationship between p53 and the GGR deficiency in melanoma, p53 transcript and protein was quantified after UVA and p53 mutation status was investigated in relation to NER levels. *p53* transcript levels were higher at all timepoints in melanocytes, significant for all cell lines at 1 and 4 h (p < 0.05) (Fig. 4a) but p53 protein was only detectable at very low levels in melanocytes at 4 and 24 h post-UVA. Interestingly, with the exception of Me4405 (p53 null) all melanoma cell lines displayed low *p53* transcript expression with slight increases at 48 h after UVA. Sk-Mel-28 has a gain of oncogenic function mutation in p53 (R273H), and along with MM200 and Mel-RM an increase in post-UVA p53 protein was detected (Fig. 4). Sk-mel-28 was the only melanoma cell line to have p53 protein detectable at baseline, before UVA. Despite large variations in p53 expression and reported activity from completely null p53 (Me4405) to gain of oncogenic function (Sk-mel-28) there was no significant differences in repair rates of CPDs across all of the melanoma cell lines.

**Fig. 5:**
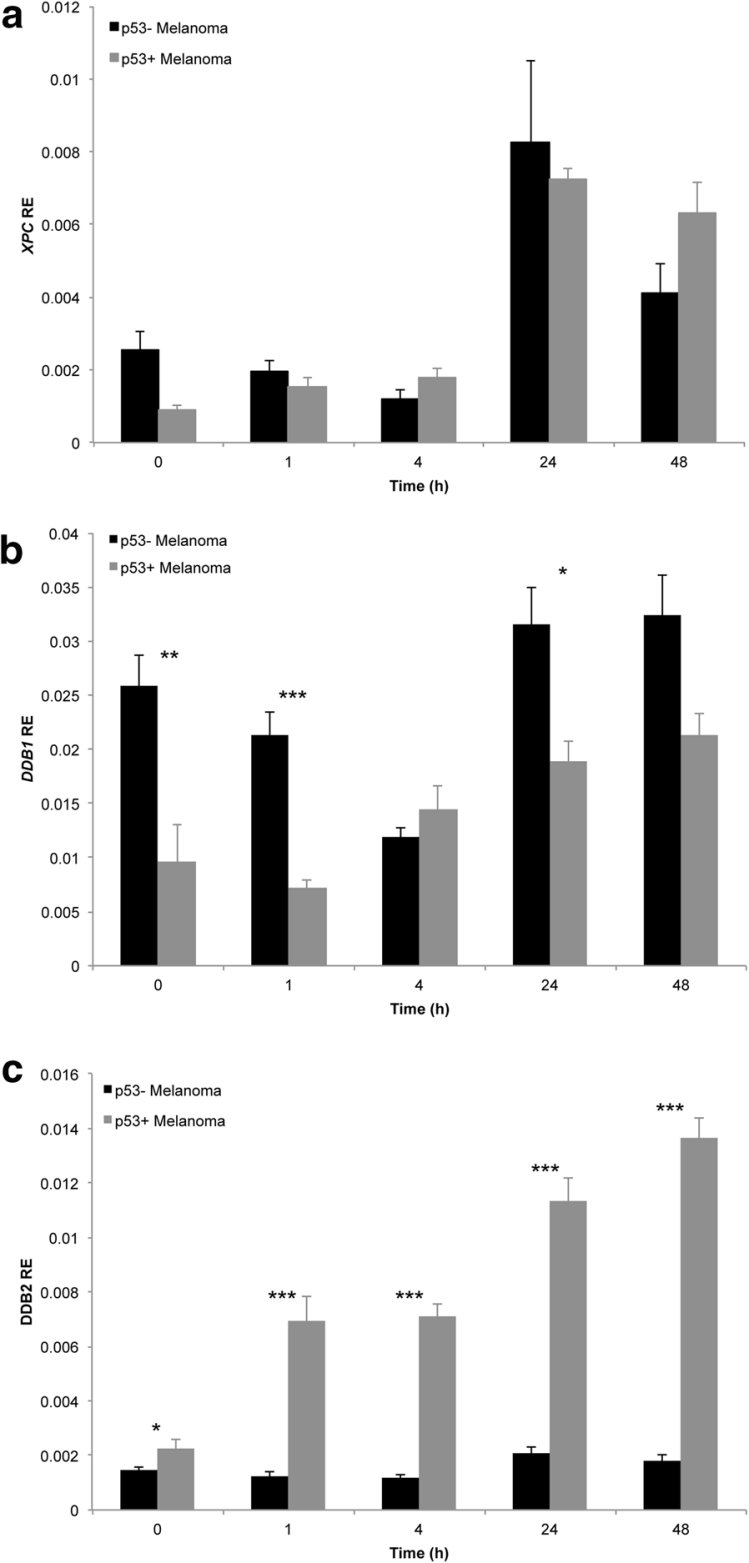
Relative expression (RE) of GGR transcripts **a** *XPC*, **b** *DDB1*, and **c** *DDB2* following UVA irradiation in p53- and p53 + melanoma cell lines. p53 mutant/null (p53−; Sk-Mel-28, Me4405) and p53 wildtype (p53+; MM200, Mel-RM melanoma cell lines were irradiated with 100 kJ/m^2^ UVA, then RE at baseline and 1, 4, 24, and 48 h post-irradiation was determined. *Points* mean of triplicate determinations of three independent experiments; *bars* ± SEM. Significant differences between groups were determined by Mann–Whitney U test. *p < 0.05, **p < 0.005, ***p < 0.000

Despite the similar CPD repair rates, significant differences in *DDB1* and *DDB2* transcript expression were observed by p53 mutation status. *DDB1* expression was significantly higher in p53 mutant/null (Sk-Mel-28, Me4405; p53− melanoma) compared to p53 wildtype (MM200, Mel-RM; p53+ melanoma) cell lines at baseline, and 1 and 24 h following UVA exposure (Fig. 5). Conversely, *DDB2* transcript expression was significantly lower in p53- melanoma cells at all time points (Fig. 5). No significant difference in *XPC* expression was observed based on p53 status (Fig. 5). The significant difference due to p53 status was not seen at the protein level for XPC, DDB1, DDB2 or in the rate of CPD removal, confirming that the GGR deficiency in melanoma is independent of p53.

In summary, Melanocytes were observed to complete repair of CPDs within 48 h following UVA exposure, with the majority of repair occurring after the 4 h time point. Preceding this, melanocytes had a marked induction of NER transcripts at 4 h, specifically, the GGR transcripts *XPC*, *DDB1*, and *DDB2*. UVA irradiation also elicited induction of GGR and p53 protein expression in melanocytes. Conversely, removal of CPD photoproducts was slower, commencing at 24 h and incomplete in melanoma cells at 48 h after UVA exposure. The GGR component XPC displayed significantly lower induction in melanoma, which was independent of p53 mutation or expression.

## Discussion

UVA constitutes the majority of solar UV (>95 %) [10] and exposure is linked to melanoma incidence [16–18], but the mechanistic relationship remains incompletely characterised. UVA induces DNA damage in the form of CPD lesions that are primarily repaired by the NER pathway; however, the NER response to UVA is uncharacterised in melanoma. Here we show that NER of UVA-induced CPDs is deficient in melanoma cells compared to normal melanocytes. We find that this is correlated with altered XPC expression, which is independent of p53 expression.

Consistent with previous reports [26, 50], UVA irradiation did not induce a detectable increase in (6-4)PP lesions. Irradiation with a solar available dose of UVA (100 kJ/m^2^) was observed to induce a similar level of CPD lesions as a high dose of UVB (600 J/m^2^). This highlights that although UVA rays are lower energy than UVB rays, the high level of environmental UVA exposure equates to a dose that induces a high level of DNA damage.

In comparison to normal melanocytes, melanoma cells displayed a delayed and attenuated DNA repair response to UVA (Fig. 2). Whilst melanocytes completed CPD repair within 48 h, more than 40 % of lesions remained in all melanoma cell lines at 48 h following irradiation. In melanocytes, the majority of repair occurred after the 4 h time point; whereas in melanoma cells the majority of repair occurred after 24 h. The relative deficiency in NER activity in melanoma cells is consistent with results reported by Belanger et al. [39], wherein it was observed that (6-4)PP repair in response to UVB and UVC was slower in melanoma cell lines compared to melanocytes. While Gaddameedhi et al. [51] reported no difference in excision repair rates between melanoma and melanocyte cell strains in response to UVC, their study did not normalise the amount of DNA damage to cell number; in contrast to Belanger et al. [39] and our study.

While CPDs have been largely considered to be produced by the direct absorption of UV energy by DNA [24], indirect production of CPDs following UVA and UVB exposure has recently been described. Premi et al. [52] have shown that UV induced reactive oxygen and nitrogen species react with melanin to produce a dioxetane intermediate that may produce CPDs in the absence of light through energy transfer to DNA. This leads to the continued production of CPDs—termed “dark CPDs”—for more than 3 h following UV exposure in melanin containing cells [52]. However, the magnitude of the dark CPD effect is variable between samples, possibly dependent on inter-individual variation in level of melanin and melanin type. Interestingly, continued production of CPDs at early time points following UVA irradiation was observed in our study in melanoma cells only. It is not clear if melanocytes also produced dark CPDs that were rapidly repaired, this requires further investigation. Irrespective of this, the melanoma cell lines displayed delayed onset of repair to 24 h, resulting in a large accumulation of CPDs.

The delayed induction of CPD repair after 24 h observed in melanoma cells corresponded with the delayed induction of GGR and *p53* transcripts in response to UVA. Despite this, melanoma cells displayed a DDB2 protein induction profile comparable to melanocytes, and two (Sk-Mel-28, Mel-RM) of four melanoma cell lines displayed induction of DDB1 similar to normal melanocytes. Given the significantly lower *DDB1* and *DDB2* transcript expression observed in melanoma cells, this suggests enhanced translation rate or protein stability of DDB1 and DDB2 in melanoma cells.

A notable difference between melanoma cell lines and melanocytes was observed for XPC protein expression. *XPC* transcript expression was significantly higher in melanocytes compared to all melanoma cell lines at 4 h. NER function is correlated with XPC expression [53], suggesting altered protein expression of XPC in melanoma may contribute to the observed reduced level of NER activity in melanoma.

Despite lower relative transcript expression, melanoma cell lines displayed a higher level of p53 protein induction compared to melanocytes. All cell lines except Me4405 (p53 null) displayed a similar p53 protein induction pattern; with expression peaking at 4 or 24 h following UVA exposure. p53 is known to display abnormal function in melanoma, and is commonly overexpressed at the protein level [40, 54]. Whether this is a potential cause or consequence of reduced GGR in melanoma requires further studies. Our study confirms increased p53 expression does not directly result in increased NER in melanoma.

Within melanoma cell lines, significant differences in GGR transcript expression were seen in relation to p53 mutation status. p53 is a known transcriptional regulator of GGR genes *DDB2* and *XPC. DDB2* expression was significantly lower in p53 mutant/null cell lines compared to p53 wildtype melanoma cell lines, at all time points. Conversely, no significant difference in *XPC* expression was observed according to p53 status, and p53 mutant/null cells displayed comparatively higher expression of *DDB1.* Suggesting that overall expression of GGR is independent of p53.

The most dominant mutational signature seen in melanoma is C to T changes due to UVR [35, 55, 56]. Further investigation of the UVR mutational signature found a higher prevalence in lowly transcribed genes, suggesting that reduced activity of the GGR component of the NER pathway is predominantly responsible for the accumulation of the UVR mutational signature in melanoma. The evidence to date, including this study suggests TCR is fully functional in melanoma. To further support this finding we previously identified significantly lower mRNA expression of all the GGR related genes in melanoma cell lines compared to melanocytes [57] after cisplatin treatment, but there was no difference in TCR response. Taken together, the results of all these studies indicate that GGR is deficient in melanoma, a finding that is largely overlooked in clinical studies of the disease.

The finding of reduced NER function in melanoma has important clinical implications. In the childhood skin cancer disorder xeroderma pigmentosum (XP) DNA damage accumulates rapidly after UV-light exposure that is associated with the development of many skin cancers on sunlight exposed areas of skin. A distinguishing feature of XP resulting from GGR mutations is mutation accumulation across most of the genome, whereas TCR deficient disorders, such as Cockayne’s syndrome, involve only actively transcribed genes [58]. The consequence of involvement of the whole genome is an increased risk of developing cancer in patients with XP, whereas patients with Cockayne’s syndrome predominantly display neurological symptoms with no increase in cancer incidence [58]. This is directly attributable to causative disease mutations being present in GGR genes, XPC and DDB2 (XPE) in patients with XP, but not in the transcription coupled repair (TCR) genes CSA and CSB in Cockayne’s syndrome. Similarly, mutations in genes involved in the mismatch repair (MMR) pathway result in hereditary non-polyposis colorectal cancer (HNPCC) or Lynch Syndrome which is characterised primarily by increased risk of colorectal, endometrial and other cancers [59]. Recent studies have reported an increased response to anti-PD1 immunotherapy in MMR-deficient cancers, suggesting that genomic instability plays a major role in the response [60]. Therefore, the deficiency in GGR reported in this study is likely to directly contribute to increased mutations across the melanoma genome and the response to anti-PD1 immunotherapy.

DDB2 and XPC have been linked to the induction of apoptosis following UVB and cisplatin respectively, through mechanisms independent of p53 [48, 49]. Consistent with this, DDB2-deficient cells have been shown to be resistant to UVB and cisplatin induced apoptosis [61, 62]. Therefore, reduced GGR may not only lead to the high mutation signature of melanoma, but may also facilitate survival of mutant cells and enhance chemoresistance.

## Conclusions

This is the first study to show that an easily obtainable dose of UVA is sufficient to induce CPD formation followed by rapid repair and GGR transcript and protein expression increases in normal melanocytes. The same GGR response was not observed in melanoma, resulting in CPD repair deficiency and an accumulation of damage 48 h after UVA. This study is the first to provide evidence that NER deficiency in melanoma is responsible for the high C > T mutation load as a result of UVA exposure and has implications for resistance to DNA-damaging chemotherapy such as cisplatin.

## References

[1] Kong Y, Kumar SM, Xu X (2010). Molecular pathogenesis of sporadic melanoma and melanoma-initiating cells. Arch Pathol Lab Med.

[2] Tsao H, Chin L, Garraway LA, Fisher DE (2012). Melanoma: from mutations to medicine. Genes Dev.

[3] Wilkins DK, Nathan PD (2009). Therapeutic opportunities in noncutaneous melanoma. Ther Adv Med Oncol.

[4] Flaherty KT, Robert C, Hersey P, Nathan P, Garbe C, Milhem M (2012). Improved survival with MEK inhibition in BRAF-mutated melanoma. N Engl J Med.

[5] Flaherty KT, Puzanov I, Kim KB, Ribas A, McArthur GA, Sosman JA (2010). Inhibition of mutated, activated BRAF in Metastatic melanoma. N Engl J Med.

[6] Shtivelman E, Davies MQ, Hwu P, Yang J, Oren M, Flaherty KT (2014). Pathways and therapeutic targets in melanoma. Oncotarget.

[7] Tsai KK, Daud AI (2015). Nivolumab plus ipilimumab in the treatment of advanced melanoma. J Hematol Oncol.

[8] Armstrong BK, Kricker A (1993). How much melanoma is caused by sun exposure?. Melanoma Res.

[9] Maverakis E, Miyamura Y, Bowen MP, Correa G, Ono Y, Goodarzi H (2010). Light, including ultraviolet. J Autoimmun.

[10] Parisi AV, Kimlin MG (2000). Estimate of annual ultraviolet-A exposures in cars in Australia. Radiat Prot Dosimetry.

[11] Bennett DC, Bennett DC (2008). Ultraviolet wavebands and melanoma initiation. Pigment Cell Melanoma Res.

[12] Diffey BL (1980). Ultraviolet radiation physics and the skin. Phys Med Biol.

[13] Diffey BL, Farr PM (1991). Quantitative aspects of ultraviolet erythema. Clin Phys Physiol Meas.

[14] He Y-Y, Huang J-L, Sik RH, Liu J, Waalkes MP, Chignell CF (2004). Expression profiling of human keratinocyte response to ultraviolet A: implications in apoptosis. J Invest Dermatol.

[15] Koch-Paiz CA, Amundson SA, Bittner ML, Meltzer PS, Fornace AJ (2004). Functional genomics of UV radiation responses in human cells. Mutat Res.

[16] Garland CF, Garland FC, Gorham ED (2003). Epidemiologic evidence for different roles of ultraviolet A and B radiation in melanoma mortality rates. Ann Epidemiol.

[17] Greinert R (2009). Skin cancer: new markers for better prevention. Pathobiology.

[18] Moan J, Dahlback A, Setlow RB (1999). Epidemiological support for an hypothesis for melanoma induction indicating a role for UVA radiation. Photochem Photobiol.

[19] Gallagher RP, Spinelli JJ, Lee TK (2005). Tanning beds, sunlamps, and risk of cutaneous malignant melanoma. Cancer Epidemiol Biomarkers Prev.

[20] Hirst N, Gordon L, Gies P, Green AC (2009). Estimation of avoidable skin cancers and cost-savings to government associated with regulation of the solarium industry in Australia. Health Policy.

[21] International Agency for Research on Cancer Working Group on artificial ultraviolet l, skin c (2007). The association of use of sunbeds with cutaneous malignant melanoma and other skin cancers: a systematic review. Int J Cancer.

[22] Ting W, Schultz K, Cac NN, Peterson M, Walling HW (2007). Tanning bed exposure increases the risk of malignant melanoma. Int J Dermatol.

[23] El Ghissassi F, Baan R, Straif K, Grosse Y, Secretan B, Bouvard V (2009). A review of human carcinogens–part D: radiation. Lancet Oncol.

[24] Ichihashi M, Ueda M, Budiyanto A, Bito T, Oka M, Fukunaga M (2003). UV-induced skin damage. Toxicology.

[25] Sutherland JC, Griffin KP (1981). Absorption spectrum of DNA for wavelengths greater than 300 nm. Radiat Res.

[26] Besaratinia A, Yoon J-I, Schroeder C, Bradforth SE, Cockburn M, Pfeifer GP (2011). Wavelength dependence of ultraviolet radiation-induced DNA damage as determined by laser irradiation suggests that cyclobutane pyrimidine dimers are the principal DNA lesions produced by terrestrial sunlight. Faseb J.

[27] Batty DP, Wood RD (2000). Damage recognition in nucleotide excision repair of DNA. Gene.

[28] Greenman C, Stephens P, Smith R, Dalgliesh GL, Hunter C, Bignell G (2007). Patterns of somatic mutation in human cancer genomes. Nature.

[29] Cleaver JE, Lam ET, Revet I (2009). Disorders of nucleotide excision repair: the genetic and molecular basis of heterogeneity. Nat Rev Genet.

[30] Hoeijmakers JH (2001). Genome maintenance mechanisms for preventing cancer. Nature.

[31] Nouspikel T (2009). DNA repair in mammalian cells: Nucleotide excision repair: variations on versatility. Cell Mol Life Sci.

[32] Marteijn JA, Lans H, Vermeulen W, Hoeijmakers JHJ (2014). Understanding nucleotide excision repair and its roles in cancer and ageing. Nat Rev Mol Cell Biol.

[33] Bowden NA (2014). Nucleotide excision repair: why is it not used to predict response to platinum-based chemotherapy?. Cancer Lett.

[34] Stoyanova T, Roy N, Kopanja D, Bagchi S, Raychaudhuri P (2009). DDB2 decides cell fate following DNA damage. Proc Natl Acad Sci USA.

[35] Pleasance ED, Cheetham RK, Stephens PJ, McBride DJ, Humphray SJ, Greenman CD (2010). A comprehensive catalogue of somatic mutations from a human cancer genome. Nature.

[36] Courdavault S, Baudouin C, Charveron M, Canguilhem B, Favier A, Cadet J (2005). Repair of the three main types of bipyrimidine DNA photoproducts in human keratinocytes exposed to UVB and UVA radiations. DNA Repair (Amst).

[37] Tewari A, Sarkany RP, Young AR (2012). UVA1 induces cyclobutane pyrimidine dimers but not 6-4 photoproducts in human skin *in vivo*. J Invest Dermatol..

[38] Bowden NA, Ashton KA, Avery-Kiejda KA, Zhang XD, Hersey P, Scott RJ (2010). Nucleotide excision repair gene expression after Cisplatin treatment in melanoma. Cancer Res.

[39] Belanger F, Rajotte V, Drobetsky EA (2014). A majority of human melanoma cell lines exhibits an S phase-specific defect in excision of UV-induced DNA photoproducts. PLoS One.

[40] Avery-Kiejda KA, Zhang XD, Adams LJ, Scott RJ, Vojtesek B, Lane DP (2008). Small molecular weight variants of p53 are expressed in human melanoma cells and are induced by the DNA-damaging agent cisplatin. Clin Cancer Res.

[41] Zhang XD, Franco AV, Myers K, Gray C, Nguyen T, Hersey P (1999). Relation of TNF-related apoptosis inducing ligand (TRAIL) receptor and FLICE-inhibitory protein expression to TRAIL-induced apoptosis of melanoma. Cancer Res.

[42] Nishinaga M, Kurata R, Onishi K, Kuriyama K, Wakasugi M, Matsunaga T (2012). Establishment of a microplate-formatted cell-based immunoassay for rapid analysis of nucleotide excision repair ability in human primary cells. Photochem Photobiol.

[43] Bowden NA, Weidenhofer J, Scott RJ, Schall U, Todd J, Michie PT (2006). Preliminary investigation of gene expression profiles in peripheral blood lymphocytes in schizophrenia. Schizophr Res.

[44] Ford JM, Hanawalt PC (1997). Expression of wild-type p53 is required for efficient global genomic nucleotide excision repair in UV-irradiated human fibroblasts. J Biol Chem.

[45] Smith ML, Ford JM, Hollander MC, Bortnick RA, Amundson SA, Seo YR (2000). p53-mediated DNA repair responses to UV radiation: studies of mouse cells lacking p53, p21, and/or gadd45 genes. Mol Cell Biol.

[46] Krzeszinski JY, Choe V, Shao J, Bao X, Cheng H, Luo S (2014). XPC promotes MDM2-mediated degradation of the p53 tumor suppressor. Mol Cell Biol.

[47] Itoh T, O’Shea C, Linn S, Itoh T, O’Shea C, Linn S (2003). Impaired regulation of tumor suppressor p53 caused by mutations in the xeroderma pigmentosum DDB2 gene: mutual regulatory interactions between p48(DDB2) and p53. Mol Cell Biol.

[48] Wang QE, Han C, Zhang B, Sabapathy K, Wani AA, Wang Q-E (2012). Nucleotide excision repair factor XPC enhances DNA damage-induced apoptosis by downregulating the antiapoptotic short isoform of caspase-2. Cancer Res.

[49] Stoyanova T, Roy N, Kopanja D, Bagchi S, Raychaudhuri P (2009). DDB2 decides cell fate following DNA damage. Proc Natl Acad Sci USA.

[50] Cortat B, Garcia CCM, Quinet A, Schuch AP, de Lima-Bessa KM, Menck CFM (2013). The relative roles of DNA damage induced by UVA irradiation in human cells. Photochem Photobiol Sci.

[51] Gaddameedhi S, Kemp MG, Reardon JT, Shields JM, Smith-Roe SL, Kaufmann WK (2010). Similar nucleotide excision repair capacity in melanocytes and melanoma cells. Cancer Res.

[52] Premi S, Wallisch S, Mano CM, Weiner AB, Bacchiocchi A, Wakamatsu K (2015). Photochemistry. Chemiexcitation of melanin derivatives induces DNA photoproducts long after UV exposure. Science.

[53] Sugasawa K (2006). UV-induced ubiquitylation of XPC complex, the UV-DDB-ubiquitin ligase complex, and DNA repair. J Mol Histol.

[54] Avery-Kiejda KA, Bowden NA, Croft AJ, Scurr LL, Kairupan CF, Ashton KA (2011). P53 in human melanoma fails to regulate target genes associated with apoptosis and the cell cycle and may contribute to proliferation. BMC Cancer.

[55] Wei X, Walia V, Lin JC, Teer JK, Prickett TD, Gartner J (2011). Exome sequencing identifies GRIN2A as frequently mutated in melanoma. Nat Genet.

[56] Turajlic S, Furney SJ, Lambros MB, Mitsopoulos C, Kozarewa I, Geyer FC et al. Whole genome sequencing of matched primary and metastatic acral melanomas. Genome Res. 2012; Advanced online publication (December 19, 2011). 10.1101/gr.125591.111 PMC3266028 22183965

[57] Bowden NA, Ashton KA, AveryKiejda KA, Zhang XD, Hersey P, Scott RJ (2010). Nucleotide excision repair gene expression after cisplation treatment in melanoma. Cancer Res..

[58] Cleaver JE (2005). Cancer in xeroderma pigmentosum and related disorders of DNA repair. Nat Rev Cancer.

[59] Lynch HT, Lynch PM, Lanspa SJ, Snyder CL, Lynch JF, Boland CR (2009). Review of the Lynch syndrome: history, molecular genetics, screening, differential diagnosis, and medicolegal ramifications. Clin Genet.

[60] Lin AY, Lin E (2015). Programmed death 1 blockade, an Achilles heel for MMR-deficient tumors?. J Hematol Oncol.

[61] Barakat BM, Wang Q-E, Han C, Milum K, Yin D-T, Zhao Q (2010). Overexpression of DDB2 enhances the sensitivity of human ovarian cancer cells to cisplatin by augmenting cellular apoptosis. Int J Cancer.

[62] Itoh T (2006). Xeroderma pigmentosum group E and DDB2, a smaller subunit of damage-specific DNA binding protein: proposed classification of xeroderma pigmentosum, Cockayne syndrome, and ultraviolet-sensitive syndrome. J Dermatol Sci.

